# Recurrence Risk Evaluation in Patients with Papillary Thyroid Carcinoma: Multicenter Machine Learning Evaluation of Lymph Node Variables

**DOI:** 10.3390/cancers15020550

**Published:** 2023-01-16

**Authors:** Sung-Woo Jang, Jae-Hyun Park, Hae-Rim Kim, Hyeong-Ju Kwon, Yu-Mi Lee, Suck-Joon Hong, Jong-Ho Yoon

**Affiliations:** 1 Department of Surgery, National Medical Center, Seoul 04564, Republic of Korea; 2 Division of Thyroid-Endocrine Surgery, Department of Surgery, Wonju Severance Christian Hospital, Yonsei University Wonju College of Medicine, Wonju 26426, Republic of Korea; 3 College of Natural Science, School of Statistics, University of Seoul, Seoul 02504, Republic of Korea; 4 Department of Pathology, Wonju Severance Christian Hospital, Yonsei University Wonju College of Medicine, Wonju 26426, Republic of Korea; 5 Department of Surgery, Asan Medical Center, Ulsan University School of Medicine, Seoul 05505, Republic of Korea; 6 Department of Surgery, Uijeongbu Eulji Medical Center, Eulji University School of Medicine, Uijeongbu 11759, Republic of Korea

**Keywords:** papillary, thyroid cancer, lymph node, risk, recurrence, recurrence-free survival

## Abstract

**Simple Summary:**

The analytic appropriateness and general applicability of lymph node-related risk factors used to predict long-term outcomes in patients with papillary thyroid carcinoma need to be validated. This study aimed to assess detailed lymph node-related risk factors and suggest new risk categories. In the present study, using the K-means clustering algorithm, we determined new cutoffs for lymph node variables besides extranodal extension: 0.2 cm and 1.1 cm for the maximal diameter of metastatic lymph node foci, 4 and 13 for the number of metastatic lymph nodes, and 0.28 and 0.58 for the metastatic lymph node ratio; new lymph node risk categories were suggested. The recurrence-free survival curves of each subgroup classified by these newly determined cutoffs showed significant differences. These newly developed risk categories might be considered when redefining risk stratification or staging systems.

**Abstract:**

Background: Lymph node (LN)-related risk factors have been updated to predict long-term outcomes in patients with papillary thyroid carcinoma (PTC). However, those factors’ analytic appropriateness and general applicability must be validated. This study aimed to assess LN-related risk factors, and suggest new LN-related risk categories. Methods: This multicenter observational cohort study included 1232 patients with PTC with N1 disease treated with a total thyroidectomy and neck dissection followed by radioactive iodine remnant ablation. Results: The median follow-up duration was 117 months. In the follow-up period, structural recurrence occurred in 225 patients (18.3%). Among LN-related variables, the presence of extranodal extension (*p* < 0.001), the maximal diameter of metastatic LN foci (*p* = 0.029), the number of retrieved LNs (*p* = 0.003), the number of metastatic LNs (*p* = 0.003), and the metastatic LN ratio (*p* < 0.001) were independent risk factors for structural recurrence. Since these factors showed a nonlinear association with the hazard ratio of recurrence-free survival (RFS) rates, we calculated their optimal cutoff values using the K-means clustering algorithm, selecting 0.2 cm and 1.1 cm for the maximal diameter of metastatic LN foci, 4 and 13 for the number of metastatic LN, and 0.28 and 0.58 for the metastatic LN ratio. The RFS curves of each subgroup classified by these newly determined cutoff values showed significant differences (*p* < 0.001). Each LN risk group also showed significantly different RFS rates from the others (*p* < 0.001). Conclusions: In PTC patients with an N1 classification, our novel LN-related risk estimates may help predict long-term outcomes and design postoperative management and follow-up strategies. After further validation studies based on independent datasets, these risk categories might be considered when redefining risk stratification or staging systems.

## 1. Introduction

Although papillary thyroid carcinoma (PTC) typically has an indolent oncologic behavior and an excellent prognosis, structural recurrence during postoperative follow-up may occur [1,2]. Various clinicopathological variables are associated with structural recurrence, recurrence-free survival (RFS), or disease-specific survival (DSS), and are used in international guidelines and staging or scoring systems to stratify the risk of structural recurrence [1,3,4,5,6,7,8,9,10,11,12,13,14,15,16,17]. 

Lymph node (LN) metastasis and LN-related variables, such as the number or ratio of metastatic LNs (MLNs), maximal diameter of metastatic LN foci, and extranodal extension, have been investigated as risk factors for target outcomes [1,3,4,5,6,7,8,9,10,11,12,13,14,15,16,17,18]. The 2015 American Thyroid Association (ATA) guidelines introduced a new risk stratification system based on the number of MLNs (five for low- and intermediate-risk categories) and the maximal diameter of MLN foci (0.2 cm for low- and intermediate-risk categories, and 3.0 cm for intermediate- and high-risk categories) [1]. Although central compartment LN metastases are prevalent in patients with PTC, most international guidelines do not recommend a prophylactic central compartment node dissection (CCND) [1,19,20]. Therefore, the studies identifying detailed LN-related risk factors, particularly for low- and intermediate-risk categories, must have been only from institutions where prophylactic CCND was preferred and might have been limited. Most management and follow-up strategies in patients with PTC are based on individualized estimates of the risk of structural recurrence or RFS rates. Therefore, it is crucial to examine the evidence for the use of existing LN-related risk factors and validate their generalizability.

This study aimed to redefine LN-related risk categories by determining the optimal cutoff values of LN-related variables for RFS rates and to analyze clinicopathological risk factors for structural recurrence in PTC patients with pathological N1 (pN1) who underwent total thyroidectomy (TT) and neck dissection followed by radioactive iodine (RAI) remnant ablation.

## 2. Materials and Methods

### 2.1. Study Population

This observational cohort study included a total of 1232 PTC patients with pN1 who underwent TT and neck LN dissection as an initial surgical procedure and subsequent RAI remnant ablation at Wonju Severance Christian Hospital (Wonju, Korea) (*n* = 225) and Asan Medical Center (Seoul, Korea) (*n =* 1007) between 2000 and 2010. Exclusion criteria were as follows: (1) distant metastases detected at initial presentation or within 12 months after initial surgery, (2) patients without a minimum follow-up of 2 years after initial treatment, and (3) patients who underwent thyroid lobectomy. 

The clinicopathological characteristics of patients and risk factors associated with structural recurrence were assessed. The LN-related variables included in the statistical analyses were pN1 classification, the number of retrieved and MLNs, the MLN ratio (defined as the number of metastatic LNs divided by the number of retrieved LNs), the maximal diameter of MLN foci, and extranodal extension. Multivariate analysis was used to confirm which LN-related variables were significant. Next, if the significant LN-related variables were continuous, we evaluated the linearity of the association between the variables and the hazard ratio (HR) of RFS rates to identify the statistical method to be used to determine the optimal LN-related variable cutoffs for the stratification of patients by target outcome. 

### 2.2. Surgical Strategy

During the study period, we performed TT for almost all patients with PTC based on the 2009 American Thyroid Association guidelines and preferred prophylactic CCND, at a minimum unilateral CCND, even in the absence of suspicious LNs. We performed bilateral CCND only in patients with bilateral PTC or suspicious LNs on the contralateral side. Additionally, we performed lateral neck dissection only in patients with biopsy-proven lateral LN metastasis.

### 2.3. Radioactive Iodine Remnant Ablation Protocol

Radioactive iodine (RAI) remnant ablation was performed 4–6 weeks after the initial surgery with a thyroid-stimulating hormone (TSH) level higher than 30 mIU/L following thyroid hormone withdrawal or recombinant human TSH administration (2). An RAI activity of 1110 MBq was administered to patients with a multifocal tumor and/or a tumor >1.0 cm without extrathyroidal extension (ETE). An RAI activity of 2960–3700 MBq was administered to patients with any tumor measuring <4.0 cm with ETE and an RAI activity of 5550 MBq was administered to patients with a tumor measuring ≥4.0 cm with or without positive surgical resection margins. At the time of remnant ablation, the serum-stimulated thyroglobulin (Tg) level and the anti-Tg antibody (Ab) level were simultaneously measured. A post-ablation whole-body scan (WBS) was obtained 2 days after the administration of iodine-131.

### 2.4. Postoperative Follow-Up Protocol

All patients were followed at the outpatient clinic every 6 months or year, checking serum Tg/anti-Tg Ab levels and neck ultrasonography during the follow-up period. If the suppressed or stimulated Tg level at 6–12 months after remnant ablation was ≥0.2 ng/mL or ≥1 ng/mL, but neck ultrasonography showed no evidence of disease, 18F-deoxyglucose positron emission tomography (FDG-PET) or chest computed tomography (CT) imaging was considered to rule out the persistent or remnant disease. 

We defined structural recurrence as newly identified malignant tissue after a period of no evidence of disease for a minimum of 1 year after initial treatment, confirmed by fine-needle aspiration cytology (FNAC) or surgical biopsy for locoregional lesions and imaging studies for distant lesions.

### 2.5. Statistical Analysis

**Figure 1:**
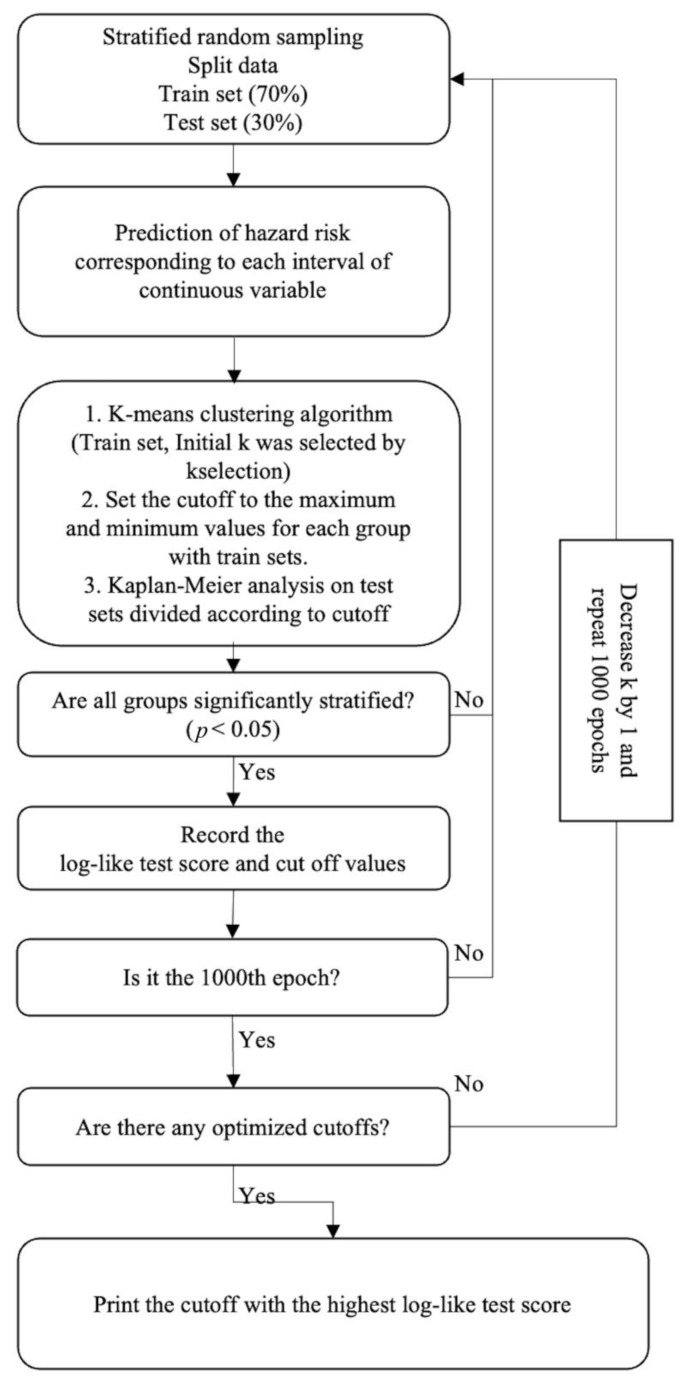
K-means clustering algorithm.

Descriptive statistics were used to characterize baseline characteristics. Continuous variables are presented as mean ± standard deviation (SD) or median and interquartile range (IQR), and categorical variables as absolute numbers and percentages (%). The *t*-test and the Mann–Whitney U test were used to compare continuous variables between the two groups. The Chi-squared and Fisher exact tests were used to assess categorical variables. Univariate and multivariate Cox proportional hazard models were used to investigate the relationship between the variables and structural recurrence and to calculate HRs with 95% confidence intervals (CIs). Since the number of retrieved LNs and MLNs, and the MLN ratio were directly related, these were analyzed using separate models. Variance inflation factors (VIF) were used to assess multicollinearity between the independent variables. VIF values exceeding 10 indicated that the relevant variable was highly correlated with the other variables in the model. Restricted cubic spline analysis in the Cox proportional hazard model was used to confirm the linearity of the association between each significant continuous variable and the HR for the RFS rate [21,22]. The K-means clustering algorithm (Supplementary Materials, File S1) was adopted to determine the optimal cutoff values of LN-related continuous variables for the target outcome (Figure 1) [23]. 

Two-sided *p*-values of <0.05 were considered significant. Statistical analyses and visualizations were conducted using R version 4.0.4 (R Foundation for Statistical Computing, Vienna, Austria http://www.R-project.org/, accessed on 15 October 2022).

## 3. Results

### 3.1. Baseline Demographic Characteristics

The clinicopathological characteristics of all patients are summarized in Table 1. The median follow-up duration was 117 months (53–134 months). 

Two hundred twenty-five patients (18.3%) had a structural recurrence. Of these, 199 patients (16.2%) showed locoregional recurrence, 15 patients (1.2%) showed distant metastasis, and 11 patients (0.9%) showed both locoregional recurrence and distant metastasis. Locoregional recurrence sites were the central compartment in 31 patients, previously undissected lateral LNs in 182 patients, and combined central and lateral LNs in 3 patients. Distant metastases were the lung in 15 patients, lung and bone in 3 patients, bone in 1 patient, and multiple organs in 7 patients. Thirteen patients (1.1%) died from PTC during the follow-up period. All patients with locoregional structural recurrence underwent reoperation, and those with distant metastasis underwent a therapeutic high-dose RAI treatment.

### 3.2. Risk Factor Analysis for Structural Recurrence

Multivariate analysis identified the following as independent risk factors for structural recurrence: male sex (HR, 1.57 [CI, 1.08–2.28]; *p* = 0.017), primary tumor size (HR, 1.27 [CI, 1.15–1.41]; *p* < 0.001), multifocality (HR, 1.81 [CI, 1.16–2.82]; *p* = 0.009), presence of extranodal extension (HR, 1.93 [CI, 1.33–2.81]; *p* < 0.001), maximal diameter of MLN foci (HR, 1.36 [CI, 1.03–1.79]; *p* = 0.029), number of retrieved LNs (HR, 0.97 [CI, 0.95–0.99]; *p* = 0.003), number of MLNs (HR, 1.06 [CI, 1.02–1.11]; *p* = 0.003), and MLN ratio (HR, 3.28 [CI, 1.54–3.94]; *p* < 0.001) (Table 2). In addition, pathologic N classification was not an independent risk factor for structural recurrence (*p* = 0.521) (Table 2). VIF values for all variables ranging from 1.070 to 3.122 showed no multicollinearity (Table 2).

### 3.3. Determination of the Optimal Cutoff Values of LN-Related Risk Factors for RFS and Re-Analysis of Risk Factors for Structural Recurrence

**Figure 2:**
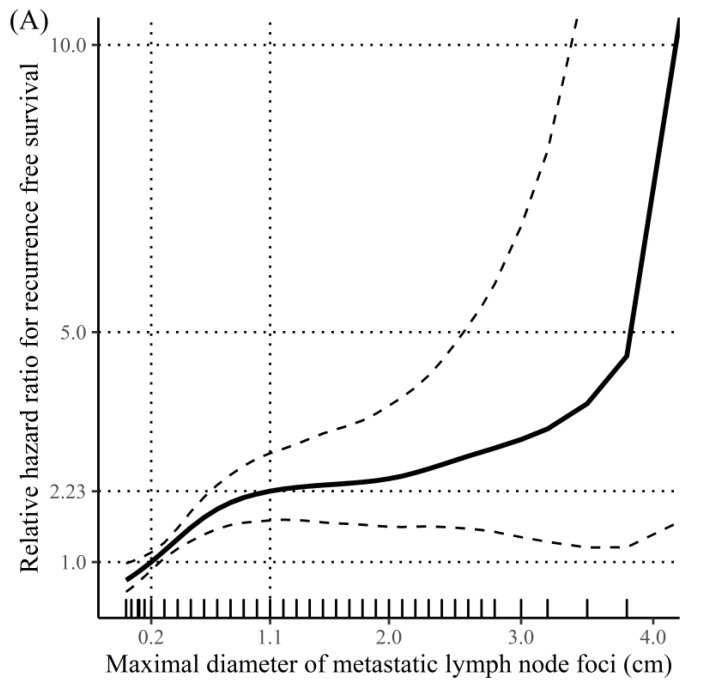

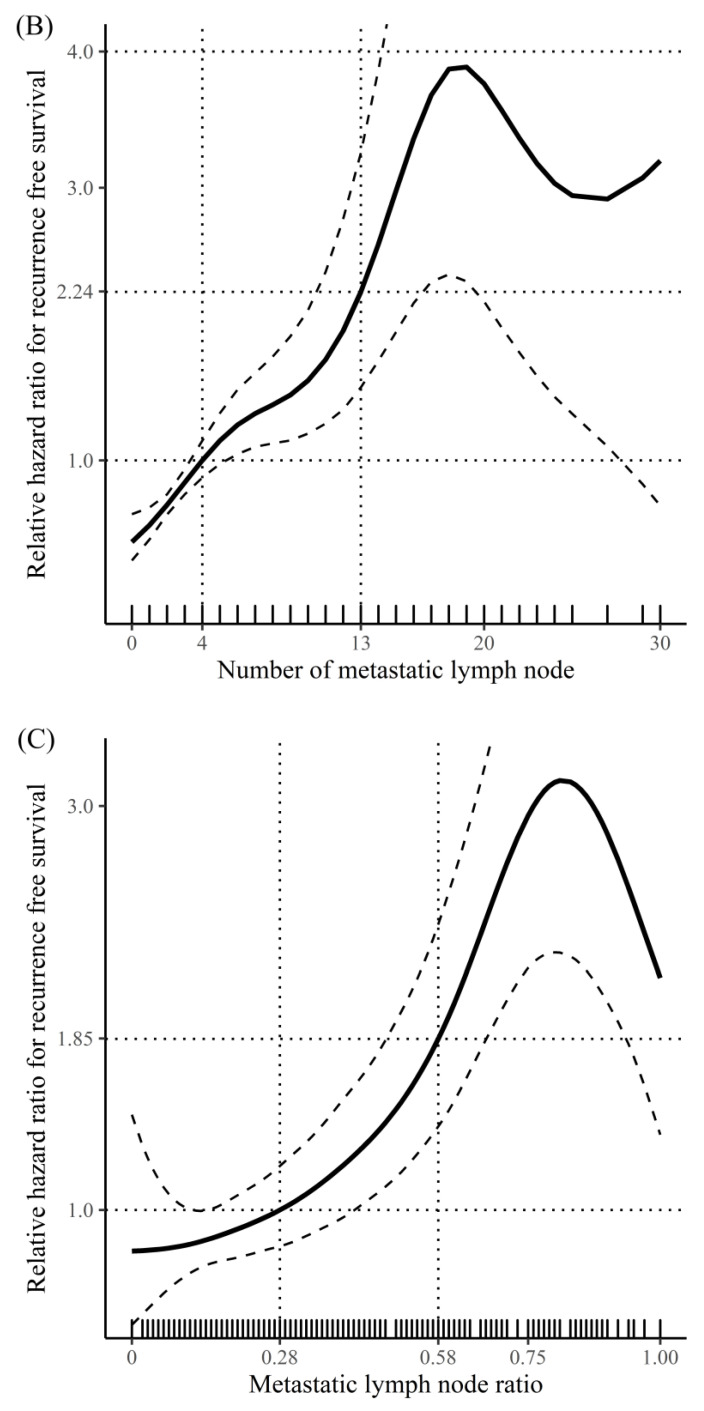
Restricted cubic spline graph showing nonlinear associations between lymph node (LN) -related continuous variables and hazard ratio for recurrence-free survival and the new cutoff values of LN-related risk factors generated using the K-means clustering algorithm: (**A**) maximal diameter of metastatic LN foci, (**B**) the number of metastatic LN, and (**C**) metastatic LN ratio.

The restricted cubic spline analysis showed nonlinear associations of the LN-related continuous variables with the HR for RFS (Figure 2). Based on the Kaplan–Meier analyses’ highest log-rank test score, the K-means clustering algorithm selects the optimal cutoff values of the LN-related risk factors, showing those with the most significant prognostic impact on the target outcome. The optimal cutoff values were 0.2 cm and 1.1 cm for the maximal diameter of MLN foci, 4 and 13 for the number of MLNs, and 0.28 and 0.58 for the MLN ratio (Figure 2). 

When the LN-related continuous variables were converted into categorical variables using these optimal cutoff values, the following were found to be independent risk factors for structural recurrence: maximal diameter of MLN foci >1.1 cm (HR, 2.16 [CI, 1.21–3.87]; *p* = 0.010), number of MLNs > 13 (HR, 3.41 [CI, 1.67–6.98]; *p* < 0.001), and MLN ratio > 0.58 (HR, 2.46 [CI, 1.54–3.94]; *p* < 0.001) (Table 2).

### 3.4. Comparison of Long-Term Outcome between the Subgroups Classified According to the Newly Determined Cutoff Values of LN-Related Risk Factors

The patients were categorized into three subgroups according to the newly generated values. Additionally, patients were categorized into three subgroups according to the existing cutoff values for the maximal diameter of MLN foci: ≤0.2 cm and >0.2 cm, and ≤3.0 cm and >3.0 cm. 

**Figure 3:**
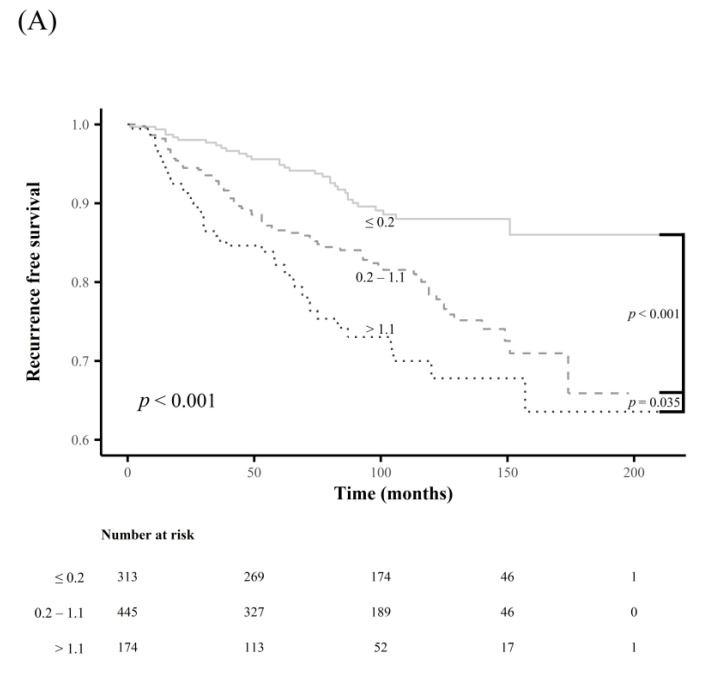

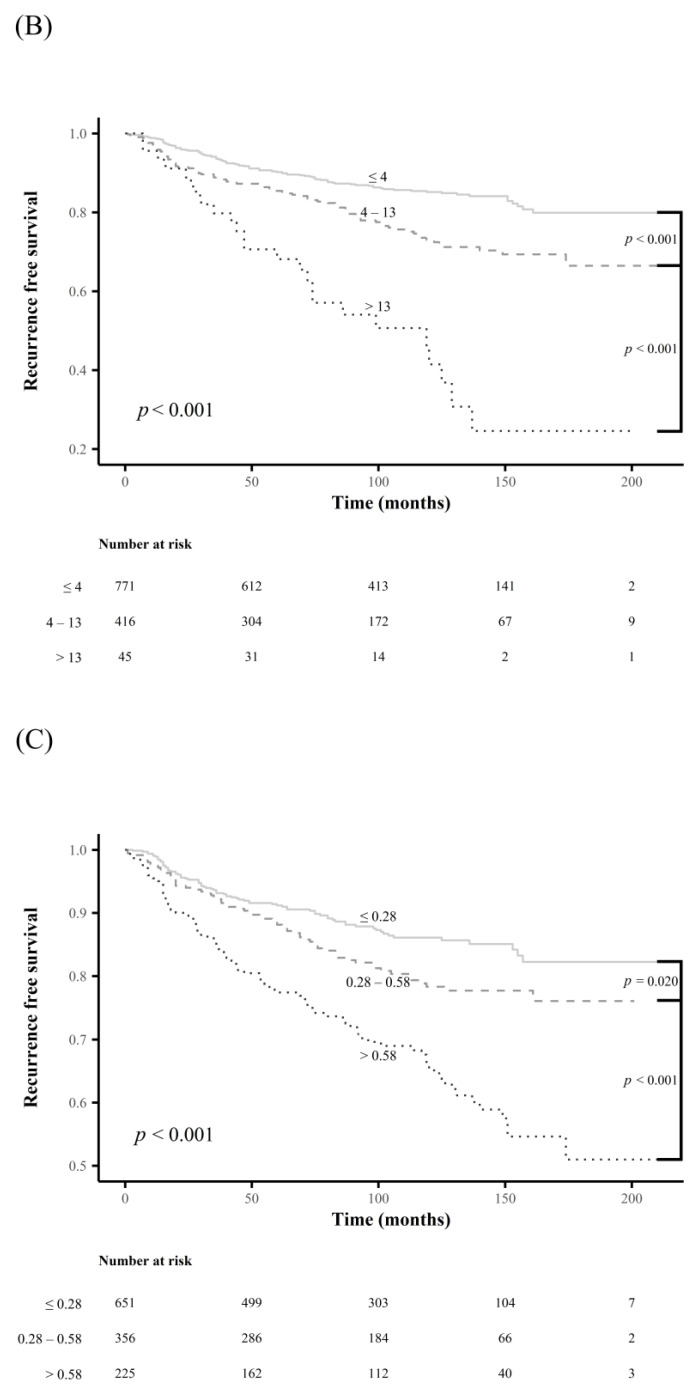

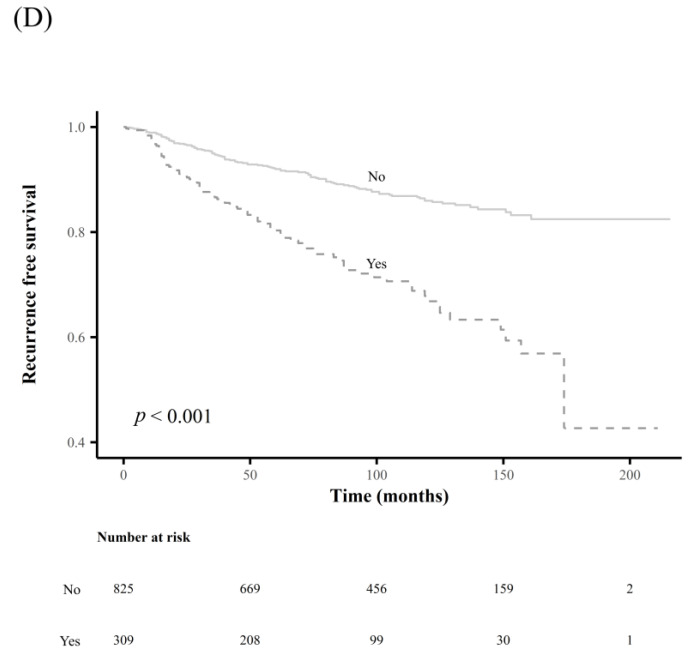
Comparison of the recurrence-free survival rates between the subgroups according to the newly determined cutoff values for LN-related risk factors: (**A**) maximal diameter of metastatic LN foci, (**B**) number of metastatic LN, (**C**) metastatic LN ratio, and (**D**) extranodal extension.

**Figure 4:**
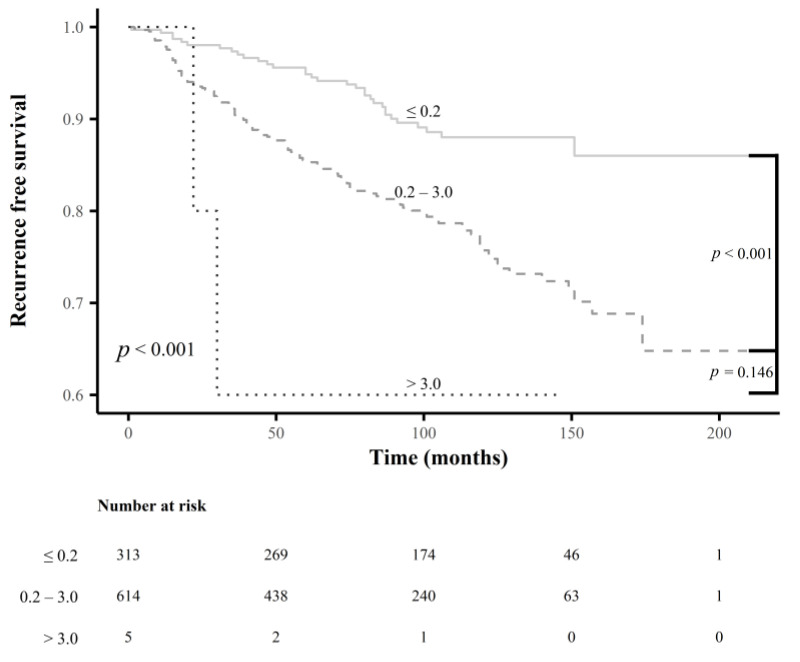
Comparison of the recurrence-free survival rates between the subgroups according to the current cutoff values for the maximal diameter of metastatic lymph node foci.

There were significant differences in RFS rates among subgroups classified using the newly determined cutoff values for each LN-related risk factor mentioned above (all *p* < 0.001) (Figure 3). Subgroups classified by the presence or absence of extranodal extension also showed a significant difference in RFS rates (*p* < 0.001) (Figure 3); however, no significant differences in RFS rates were observed for subgroups classified by the current cutoff values for the maximal diameter of MLN foci (Figure 4).

### 3.5. Proposal of New LN-Related Risk Categories for RFS

**Figure 5:**
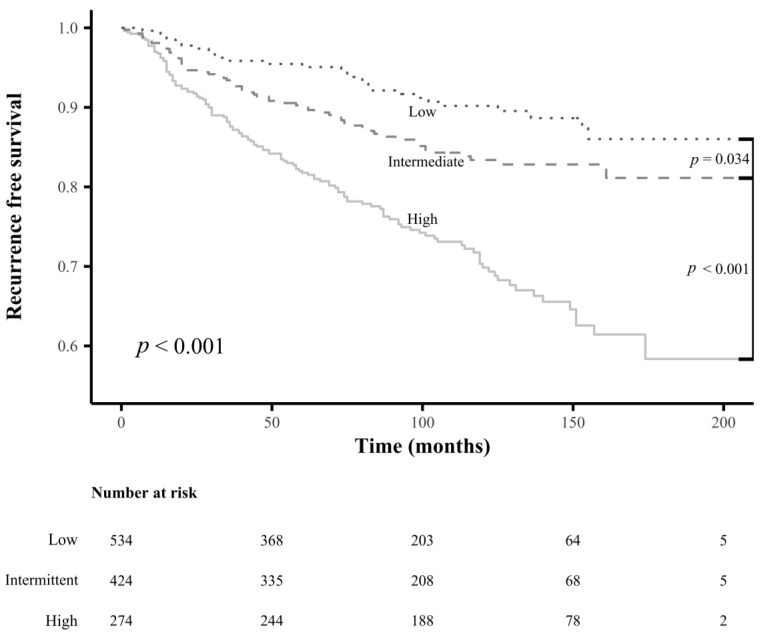
Comparison of the recurrence-free survival rates between the newly developed lymph node-related risk groups.

We have proposed novel detailed LN-related risk categories. These are summarized in Table 3. In addition, we identified a risk category for the presence of extranodal extension based on its HR compared with the HRs of the other LN-related variables classified as high-risk. There were significant differences in the RFS rates of the newly developed LN-related risk groups (*p* < 0.001) (Figure 5).

### 3.6. Comparison of Long-Term Outcome between pN0 Patients and pN1 Patients Classified into Each LN Risk Category

**Figure 6:**
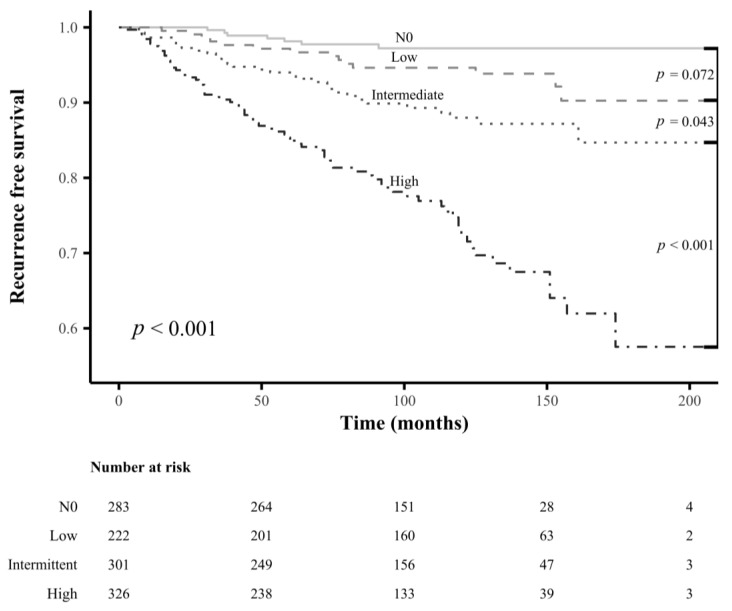
Comparison of the recurrence-free survival rates between pathologic N0 patients and pathologic N1 patients classified into the newly developed lymph node-related risk categories after 1:3 propensity score matching.

We compared the clinicopathological variables of pN1 patients with those of pN0 patients who underwent TT and neck dissection followed by RAI remnant ablation at Wonju Severance Christian Hospital during the same period (Table 4). We matched the patients with pN0 and pN1 disease for the clinicopathological variables showing significant differences by 1:3 propensity score matching (Table 5). After propensity score matching, we compared the RFS rates of pN0 patients and pN1 patients classified into each LN-related risk category; the LN-related low-risk pN1 patients did not show significantly different RFS rates from pN0 patients, but the intermediate- and high-risk pN1 patients did (Figure 6). The 5-/10-year RFS rates of patients with pN0 disease, low-, intermediate-, and high-risk pN1 groups were 98.1/97.2%, 96.7/94.6%, 94.0/88.0%, and 84.9/72.4%, respectively.

## 4. Discussion

This study proposes novel LN-related risk categories with optimal cutoff values for each detailed LN variable selected by the K-means clustering algorithm, based on a large-scale, multicenter cohort of PTC patients with pN1 who underwent TT followed by RAI remnant ablation. Considering the nonlinear associations between LN-related continuous variables and the HR of RFS, the K-means clustering algorithm generated and internally validated the optimal cutoffs for the HR of RFS, which were 0.2 cm and 1.1 cm for the maximal diameter of MLN foci, 4 and 13 for the number of MLNs, and 0.28 and 0.58 for the MLN ratio. Significant differences in the RFS rates were found among the subgroups categorized according to these cutoff values.

The current ATA initial risk stratification system estimates additional LN risk factors, namely the maximal size of MLN (with cutoffs of 0.2 cm and 3.0 cm) and the number of MLNs (with a cutoff value of five) [1]. Despite not being included in the guidelines, other LN-related risk factors, such as the number of retrieved LNs, extranodal extension, and the MLN ratio, have been found to have a prognostic impact [3,4,14,18]. We reviewed the evidence used to identify the recent LN-related risk factors. We focused on the optimal inclusion of clinicopathological variables to adjust risk factors and aimed to use the appropriate tool for modeling continuous variables and the appropriate statistical method to determine the optimal cutoff values and their numbers.

For the maximal size of MLN foci, a study involving 604 patients undergoing initial surgery for PTC > 1 cm demonstrated that large nodal metastasis ≥ 3 cm was an independent risk factor for both DSS and disease-free survival (DFS) in patients aged ≥ 50 years [8]. Another study compared the prognoses of 621 patients with N1b disease with those of 4297 patients with N0 and 125 patients with N1a, revealing that MLNs > 3 cm independently affected both DSS and DFS [10]. In these studies, there were deficiencies in the modeling tool for continuous variables and the statistical method for determining the cutoff value and its number. These studies dichotomized continuous variables with each single cutoff value, including five for the number of MLNs and 3.0 cm for the largest size of MLN, but did not specify how to determine the cutoff values. As these methods are likely to either weaken the model’s predictive ability or show a poor association between continuous variables and the target outcome, they are not recommended in the field of medicine [21,22]. Additionally, using a single cutoff may have resulted in the inadequate stratification of patients for the target outcome in models with a nonlinear association. Moreover, these studies did not include the extent of thyroidectomy as a variable in the risk factor analysis, and the latter study examined only four LN-related variables in its risk factor analysis [8,10].

Furthermore, large nodal metastasis ≥3 cm was an independent risk factor for DSS and DFS only in patients aged ≥ 50 years in the former study and only in the N1b subset in the latter study, resulting in issues with the general applicability of these results [8,10]. A further study reported that patients with MLN <0.2 cm showed significantly lower locoregional recurrence than their counterparts (5% vs. 32%, *p* = 0.015) [24]. However, this was a low-volume study that adopted the definition of LN micrometastases commonly used in breast cancer and other solid tumors but not used for thyroid cancer. A recent study based on 398 patients with pN1a classic PTC demonstrated that LN metastasis < 0.35 cm was an independent risk factor for long-term structural recurrence [3]. Although the investigators treated the variable as a continuous variable, the cutoff was determined based on a linear association between the variable and the target outcome, without considering the possibility of a nonlinear association. Therefore, the generalizability of the cutoff and its number may be challenged.

The prognostic impact of the number of MLNs on DSS and DFS has been previously investigated [8,10]. Two studies found that MLNs ≥5 were an independent risk factor for DFS only in younger patients (<50 and <55 years, respectively), but not for DFS in older patients and DSS [8,10]. Another study involving 148 consecutive patients with PTC with LN metastases examined the significance of the number of MLNs and concluded that a number of metastatic LNs >10 was an independent risk factor for persistent but not recurrent disease [11]. A previous study involving 398 patients with pN1a classic PTC analyzed the number of MLNs as a continuous variable, revealing that a number of MLNs ≥ 4 was an independent risk factor for long-term structural recurrence with significant differences in the RFS rate between the subgroups determined by the cutoff [3]. In addition to the fact that the prognostic significance of these studies was restricted to the specific patient cohort, there were further limitations concerning the modeling tool of continuous variables, the statistical method applied to determine the cutoff values, and the consideration of the possibility of a nonlinear association between variables and outcomes.

Another potential LN-related risk factor as a categorical variable is the MLN ratio. Most studies found a significant association between different MLN ratio thresholds ranging from 0.3 to 0.86 and poor RFS rates [5,6,7,9,12,14,15,16,17]. However, these studies determined only one cutoff value for the MLN ratio without considering the possibility of a nonlinear association between variables and target outcomes.

A further study based on 305 patients with PTC compared the risk of persistent/recurrent disease (PRD) between pNx, pN0, microscopic pN1, and macroscopic pN1 groups focusing on the prognostic value of microscopic LN involvement (25). The study showed that macroscopic (*p* < 0.001) and microscopic (*p* < 0.02) pN1 classification systems were independent risk factors for PRD. Furthermore, the disease-free survival rate significantly decreased from pNx (98%), pN0 (93%), and microscopic pN1 (89%) to macroscopic pN1 patients (70%) (*p* < 0.001), demonstrating the presence of microscopic LN involvement is associated with an intermediate outcome. The study also comprehensively evaluates LN variables, showing that the size of the largest MLN foci (0.2–1.0 cm and >1.0 cm), the number of MLNs (1–5 and >5), and the presence of extranodal extension were independent risk factors for PRD. Although these results were similar to ours, the study did not avoid the modeling and statistical errors mentioned above. There were further limitations concerning the small patient number (*n =* 305), short follow-up duration (4 years), and ambiguous and overlapping classification of microscopic (0.2–1.1 cm) and macroscopic (0.7–7.0 cm) LN metastases.

The present study was a large-scale, multicenter study aiming to determine a novel LN-related risk stratification system. Unlike previous studies, the present study included sufficient clinicopathological variables to analyze risk factors for long-term structural recurrence and analyzed continuous variables as a continuous variable to avoid weakening the model’s predictive ability. In addition, we excluded multicollinearity between the variables using the VIF test. As we evaluated the LN-related risk factors, we did not differentiate between N1a and N1b since the N classification was not an independent risk factor for structural recurrence, which was consistent with the 8th edition tumor, node, and metastasis staging system classifying N1a and N1b together as N1 [25]. We used the restricted cubic spline analysis to assess the association between the continuous variables and the HR for RFS and identified their nonlinear association [21,22].

Further, we determined the optimal cutoff values for generalizability and consistency with the existing risk stratification system using the K-means clustering algorithm, which automatically selected the optimal cutoff values and their numbers through machine learning [23]. The K-means clustering algorithm first determined the potential cutoff values of LN-related continuous variables which were able to stratify patients for the HR of RFS in one thousand sets of training and test groups that were randomly classified. The algorithm then selected the optimal cutoff values with the highest log-like test score for each LN-related continuous variable. This algorithm could simultaneously determine and validate the optimal cutoff values. Interestingly, some subgroups of LN risk factors sorted by their optimal cutoffs were not independent risk factors for structural recurrence, irrespective of the fact that each LN risk factor subgroup showed significantly different RFS rates. The Cox proportional hazard regression analysis used to assess risk factors imposes a stringent assumption that the association between variables and target outcomes must be linear [21,22]. Therefore, the significance of the continuous variable analyzed as a continuous variable may have been inconsistent with that found when the variable was analyzed as a categorical variable, particularly in models with a nonlinear association with the target outcome.

We acknowledge the limitations of our study, including inherent bias due to its retrospective nature and the preference of the institutions involved for prophylactic CCND, which many international guidelines do not recommend. However, prophylactic LN dissection allowed us to evaluate all of the LN variables in detail. Additionally, even though the exact causes were uncertain, the patient cohort in our study showed relatively high proportions of ETE and N1b classification, which may be indicative of aggressive disease, resulting in a relatively high recurrence rate. However, the clinicopathological status of actual patients undergoing thyroidectomy may be different from those observed in the patients in this study. Moreover, the extent of thyroidectomy and the use of RAI remnant ablation for patients with PTC have shifted toward a more conservative direction, particularly for low- and intermediate-risk patients. Therefore, it is essential to conduct further large-scale, multicenter studies to determine appropriate cutoff values for LN variables based on data from patients undergoing thyroid lobectomy only or total thyroidectomy without RAI therapy.

The present study was a large-scale, multicenter study based on adequate clinicopathological information, and all enrolled patients had a minimum follow-up duration of 2 years after the initial surgery. Additionally, we adopted a modeling tool suitable for continuous variables and used a new statistical method to assess the optimal cutoff values of continuous variables in the model with a nonlinear association with the target outcome. However, the clinical implications of our study and the generalizability of our results need to be corroborated using independent validation datasets.

## 5. Conclusions

In PTC patients with N1 classification, we propose novel LN-related risk categories, which could be used to predict long-term outcomes and design targeted postoperative management and follow-up strategies for thyroidectomy patients with N1 PTC. After additional validation studies based on independent datasets, this novel LN-related risk stratification system might be taken into consideration when risk stratification or staging systems are redefined.

## Figures and Tables

**Table 1:** Patient demographic characteristics.

Characteristics	All ^a^	NED ^a^	Recurrence ^a^	*p*
	(*n =* 1232)	(*n =* 1007)	(*n =* 225)	
Age, years, mean ± SD	44.7 ± 12.9	44.6 ± 12.2	45.3 ± 15.5	0.781
Sex				<0.001
Female	1015 (82.4)	849 (84.3)	166 (73.8)	
Male	217 (17.6)	158 (15.7)	59 (26.2)	
Primary tumor size, cm	2.0 ±1.2	1.8 ±1.1	2.5 ±1.5	<0.001
Multifocality				<0.001
No	684 (55.5)	584 (58.0)	100 (44.4)	
Yes	548 (44.5)	423 (42.0)	125 (55.6)	
Bilaterality				0.010
No	842 (68.3)	705 (70.0)	137 (60.9)	
Yes	390 (31.7)	302 (30.0)	88 (39.1)	
Microscopic extrathyroidal extension				<0.001
No	336 (27.3)	299 (29.7)	37 (16.4)	
Yes	896 (72.7)	708 (70.3)	188 (83.6)	
Lymphatic invasion				0.015
No	989 (80.3)	822 (81.6)	167 (74.2)	
Yes	243 (19.7)	185 (18.4)	58 (25.8)	
Vascular invasion				0.008
No	1126 (91.4)	931 (92.5)	195 (86.7)	
Yes	106 (8.6)	76 (7.5)	30 (13.3)	
N classification				0.119
N1a	776 (63.0)	645 (64.1)	131 (58.2)	
N1b	456 (37.0)	362 (35.9)	94 (41.8)	
Extent of LN dissection				0.022
Ipsilateral CCND	543 (44.1)	463 (46.0)	80 (35.6)	
Bilateral CCND	233 (18.9)	182 (18.1)	51 (22.7)	
Ipsilateral mRND	299 (24.3)	242 (24.0)	57 (25.3)	
Bilateral mRND	157 (12.7)	120 (11.9)	37 (16.4)	
RAI activity, MBq				<0.001
1110	51 (4.1)	46 (4.6)	5 (2.2)	
2960	282 (22.9)	246 (24.4)	36 (16.0)	
3700	390 (31.7)	328 (32.6)	62 (27.6)	
5550	509 (41.3)	387 (38.4)	122 (54.2)	
Extranodal extension				<0.001
No	923 (74.9)	782 (77.7)	141 (62.7)	
Yes	309 (25.1)	225 (22.3)	84 (37.3)	
Maximal diameter of metastatic LN foci, cm	0.7 ± 0.7	0.6 ± 0.6	0.8 ± 0.8	<0.001
Number of retrieved LN, mean ± SD	17.5 ± 13.4	17.5 ± 13.2	17.7 ± 14.0	0.783
Number of metastatic LN, mean ± SD	4.5 ±4.0	4.1 ±3.6	6.4 ±5.3	<0.001
Metastatic LN ratio, mean ± SD	0.3 ±0.3	0.3 ±0.2	0.5 ±0.3	<0.001

SD, standard deviation; LN, lymph node; NED, no evidence of disease; CCND, central compartment neck dissection; mRND, modified radical neck dissection. ^a^ Data expressed as *n* (%) unless otherwise noted

**Table 2:** Risk factor analyses for structural recurrence.

Factors	Univariate	*p*	Multivariate	*p*
Age	1.01 (1.00–1.02)	0.172	1.01 (1.00–1.02)	0.086
Sex				0.017
Female	Reference		Reference	
Male	1.97 (1.38–2.82)	<0.001	1.57 (1.08–2.28)	
Primary tumor size	1.40 (1.27–1.54)	<0.001	1.27 (1.15–1.41)	<0.001
Multifocality	1.58 (1.15–2.17)	0.004	1.81 (1.16–2.82)	0.009
Bilaterality	1.42 (1.03–1.96)	0.034	0.81 (0.51–1.29)	0.373
Microscopic extrathyroidal extension	1.70 (1.14–2.55)	0.010	1.21 (0.79–1.84)	0.381
Lymphatic invasion	1.62 (1.15–2.27)	0.005	1.01 (0.65–1.58)	0.953
Vascular invasion	2.01 (1.28–3.15)	0.003	1.39 (0.77–2.53)	0.275
N classification				0.521
N1a	Reference		Reference	
N1b	1.38 (0.99–1.93)	0.058	1.19 (0.70–2.02)	
Extranodal extension	2.91 (2.12–3.99)	<0.001	1.93 (1.33–2.81)	<0.001
Maximal diameter of metastatic LN foci	1.63 (1.34–1.97)	<0.001	1.36 (1.03–1.79)	0.029
≤0.2	Reference		Reference	
>0.2 and ≤1.1	2.14 (1.42–3.22)	<0.001	1.52 (0.97–2.37)	0.068
>1.1	3.19 (2.01–5.07)	<0.001	2.16 (1.21–3.87)	0.010
Number of retrieved LN ^a^	1.00 (0.99–1.02)	0.477	0.97 (0.95–0.99)	0.003
Number of metastatic LN ^a^	1.08 (1.05–1.11)	<0.001	1.06 (1.02–1.11)	0.003
≤4	Reference		Reference	
>4 and ≤13	1.70 (1.22–2.38)	0.002	1.21 (0.82–1.77)	0.330
>13	4.49 (2.68–7.53)	<0.001	3.41 (1.67–6.98)	<0.001
Metastatic LN ratio^a^	4.26 (2.55–7.13)	<0.001	3.28 (1.54–3.94)	<0.001
≤0.28	Reference		Reference	
>0.28 and ≤0.58	1.33 (0.89–2.00)	0.161	1.21 (0.78–1.87)	0.394
>0.58	2.89 (2.01–4.17)	<0.001	2.46 (1.54–3.94)	<0.001

LN, lymph node. ^a^ Metastatic LN ratio and the number of retrieved and metastatic LN were analyzed in separate models.

**Table 3:** Proposal of detailed lymph node-related risk categories for recurrence-free survival.

	Low	Intermediate	High
Extranodal extension	absence	-	presence
	or	or	or
Maximal diameter of metastatic LN foci, cm	≤0.2	>0.2 and ≤1.1	>1.1
	or	or	or
Number of metastatic LN	≤4	>4 and ≤13	>13
	or	or	or
Metastatic LN ratio	≤0.28	>0.28 and ≤0.58	>0.58

LN, lymph node.

**Table 4:** Comparison of demographic characteristics between pathologic N0 and N1 patients.

Characteristics	All ^a^	Pathologic N0 ^a^	Pathologic N1 ^a^	*p*
	(*n =* 1515)	(*n =* 283)	(*n =* 1232)	
Age, years, mean ± SD	34.3 ± 12.7	36.9 ± 12.0	33.7 ± 12.8	<0.001
Sex				0.019
Female	1265 (83.5)	250 (88.3)	1015 (82.4)	
Male	250 (16.5)	33 (11.7)	217 (17.6)	
Primary tumor size, cm	1.9 ± 1.2	1.4 ± 1.0	2.0 ± 1.2	<0.001
Multifocality				0.002
No	871 (57.5)	187 (66.1)	684 (55.5)	
Yes	644 (42.5)	96 (33.9)	548 (44.5)	
Bilaterality				0.165
No	1048 (69.2)	206 (72.8)	842 (68.3)	
Yes	467 (30.8)	77 (27.2)	390 (31.7)	
Microscopic extrathyroidal extension				<0.001
No	485 (32.0)	149 (52.7)	336 (27.3)	
Yes	1030 (68.0)	134 (47.3)	896 (72.7)	
Lymphatic invasion				0.040
No	1200 (79.2)	211 (74.6)	989 (80.3)	
Yes	315 (20.8)	72 (25.4)	243 (19.7)	
Vascular invasion				<0.001
No	1403 (92.6)	277 (97.9)	1126 (91.4)	
Yes	112 (7.4)	6 (2.1)	106 (8.6)	
Recurrence	232 (15.3)	7 (2.5)	225 (18.3)	<0.001
Locoregional recurrence	217 (14.3)	7 (2.5)	210 (17.0)	<0.001
Distant metastasis	27 (1.8)	1 (0.4)	26 (2.1)	0.077

SD, standard deviation. ^a^ Data expressed as *n* (%) unless otherwise noted.

**Table 5:** Comparison of demographic characteristics between pathologic N0 and N1 patients after 1:3 propensity score matching.

Characteristics	All ^a^	pN0 ^a^	Low-Risk pN1 ^a^	Intermediate-Risk pN1 ^a^	High-Risk pN1 ^a^	*p*
	(*n =* 1132)	(*n =* 283)	(*n =* 222)	(*n =* 301)	(*n =* 326)	
Age, years, mean ± SD	35.9 ± 12.3	36.9 ± 12.0	36.2 ± 10.4	35.5 ± 12.4	35.0 ± 13.6	0.221
Sex						0.119
Female	986 (87.1)	250 (88.3)	202 (91.0)	259 (86.0)	275 (84.4)	
Male	146 (12.9)	33 (11.7)	20 (9.0)	42 (14.0)	51 (15.6)	
Primary tumor size, cm	1.5 ± 0.9	1.4 ± 1.0	1.4 ± 0.8	1.5 ± 1.0	1.5 ± 0.9	0.157
Multifocality						0.249
No	704 (62.2)	187 (66.1)	141 (63.5)	186 (61.8)	190 (58.3)	
Yes	428 (37.8)	96 (33.9)	81 (36.5)	115 (38.2)	136 (41.7)	
Bilaterality						0.958
No	824 (72.8)	206 (72.8)	162 (73.0)	222 (73.8)	234 (71.8)	
Yes	308 (27.2)	77 (27.2)	60 (27.0)	79 (26.2)	92 (28.2)	
Microscopic ETE						0.976
No	465 (41.1)	149 (52.7)	115 (51.8)	154 (51.2)	166 (50.9)	
Yes	667 (58.9)	134 (47.3)	107 (48.2)	147 (48.8)	160 (49.1)	
Lymphatic invasion						0.944
No	909 (80.3)	211 (74.6)	170 (76.6)	229 (76.1)	249 (76.4)	
Yes	223 (19.7)	72 (25.4)	52 (23.4)	72 (23.9)	77 (23.6)	
Vascular invasion						0.134
No	1107 (97.8)	277 (97.9)	221 (99.5)	291 (96.7)	318 (97.5)	
Yes	25 (2.2)	6 (2.1)	1 (0.5)	10 (3.3)	8 (2.5)	
Recurrence						
Locoregional	128 (11.3)	7 (2.5)	14 (6.3)	31 (10.3)	76 (23.3)	0.000
Distant	12 (1.1)	1 (0.4)	1 (0.5)	1 (0.3)	9 (2.8)	0.010

SD, standard deviation; ETE, extrathyroidal extension. ^a^ Data expressed as *n* (%) unless otherwise noted.

## Data Availability

The data presented in this study are available upon request from the corresponding author. The data are not publicly available due to the privacy of enrolled patients.

## Supplementary Materials

The following supporting information about survival cluster analysis can be downloaded at: https://www.mdpi.com/article/10.3390/cancers15020550/s1, Supplementary File S1: Survival Cluster analysis.
