# Supplementary material for: Recurrence Risk Evaluation in Patients with Papillary Thyroid Carcinoma: Multicenter Machine Learning Evaluation of Lymph Node Variables
Source: Cancers (Basel). 2023 Jan 16;15(2):550. doi: 10.3390/cancers15020550 (PMC9856505; doi:10.3390/cancers15020550)
Supplement: Supplementary file 1 [file cancers-15-00550-s001.zip › cancers-2047560-supplementary.pdf]

Supplementary Materials: Recurrence Risk Evaluation in Patients with Papillary Thyroid Carcinoma: Multicenter Machine Learning Evaluation of Lymph Node Variables

Sung-Woo Jang, Jae-Hyun Park, Hae-Rim Kim, Hyeong-Ju Kwon, Yu-Mi Lee, Suck-Joon Hong and Jong-Ho Yoon

Survival Cluster Analysis

The K-means clustering algorithm stratifies continuous variables by determining the optimal cutoff values that maximize log-likelihood scores and predict target outcomes corresponding to continuous variables.

The unsupervised K-means clustering algorithm is an algorithm that makes numbers of K clusters to minimize the distance variance of each group in the multidimensional space of a dataset. This is a machine-learning algorithm that is used to label new imputed data after learning existing data. Although this algorithm is potent for creating a labeling prediction model, the result may vary depending on the K value (the number of subgroups classified by clustering) as an inputted parameter. Therefore, initial K was selected by K selection[1] and decreased by one until 2 for significantly dividing each subset in the survival analysis after clustering. K-means++ clustering algorithm was used to adjust the position of the initial cluster to converge to the global minimum.

In addition, to minimize the effect of outliers, the algorithm divides the train and test set by 70:30. Split train-test set by stratified random sampling method avoids allocating only one class (patients with structural recurrence in the present study) to either train or test set. The train-test split method is the most widely used in machine learning algorithms for generalized applications and preventing the overfitting of models. In the train set, cutoffs significantly stratifying all subgroups for target outcomes are obtained. Through these cutoffs, the algorithm tests that the test set is also significantly stratified. This set of processes is defined as one epoch, and the algorithm repeats 1000 epochs to find the optimal cutoff values. Based on Kaplan-Meier analyses' highest log-like test score and Cox proportional hazard model after clustering at each epoch, the optimal set of cutoffs is determined. The proportional hazards assumption is tested based on Schoenfeld residuals[2]. The cutoffs determined by the K-means clustering algorithm do not need additional validation for general applicability because the algorithm itself undergoes internal validation.
